# An RCT of three programs aimed at reducing risk factors for both eating disorders and obesity: outcomes from the Prevention Across the Spectrum study

**DOI:** 10.1186/2050-2974-2-S1-O43

**Published:** 2014-11-24

**Authors:** Simon Wilksch, Susan Paxton, Sue Byrne, S Bryn Austin, Tracey Wade

**Affiliations:** 1 School of Psychology, Flinders University, Adelaide, Australia; 2 School of Psychological Science, La Trobe University, Melbourne, Australia; 3 School of Psychology, University of Western Australia, Perth, Australia; 4 Boston Children's Hospital, Boston, USA; 5 Harvard Medical School, Boston, USA; 6 Harvard School of Public Health, Boston, USA

## Objective

To conduct an RCT of 3 school-based programs to evaluate if one or more could reduce both eating disorder and obesity risk factors. Method: N = 1,316 Grade 7 and 8 girls and boys (M age = 13.21 years) from three Australian states were randomly allocated to: Media Smart; Life Smart; Helping, Encouraging, Listening and Protecting Peers Initiative (HELPP) or control (usual school class). Risk factors were measured at baseline, post-program (5-weeks later), 6-month follow-up, and 12-month follow-up.

## Results

Media Smart girls had half the rate of onset of clinically significant concerns about shape and weight as control girls at 12-month follow-up. Media Smart and HELPP girls reported significantly lower weight concern and shape concern than Life Smart but not control girls at 12-month follow-up. Media Smart girls experienced additional benefits on eating concerns and perceived pressure to be thin compared to HELPP girls and also on levels of physical activity compared to control girls. Media Smart and HELPP boys experienced significant benefit on media internalization compared to control boys while Media Smart boys reported additional benefits including significantly lower screen time at 12-month follow-up compared to the other interventions.

## Conclusions

Media Smart was the only program to show benefit on both disordered eating and obesity risk factors.

This abstract was presented in the **Prevention & Public Health** stream of the 2014 ANZAED Conference.

